# Multiple Subpial Transection for the Treatment of Landau–Kleffner Syndrome—Review of the Literature

**DOI:** 10.3390/jcm13247580

**Published:** 2024-12-13

**Authors:** Piotr Duda, Natalia Duda, Katarzyna Kostelecka, Filip Woliński, Joanna Góra, Michał Granat, Łukasz Bryliński, Barbara Teresińska, Robert Karpiński, Wojciech Czyżewski, Jacek Baj

**Affiliations:** 1 Department of Correct, Clinical and Imaging Anatomy, Medical University of Lublin, ul. Jaczewskiego 4, 20-090 Lublin, Poland; piotr.duda.1257@gmail.com (P.D.); dudaa.natalia@gmail.com (N.D.); asiagora977@gmail.com (J.G.); michal.granat.mg@gmail.com (M.G.); 2 Department of Forensic Medicine, Medical University of Lublin, Jaczewskiego 8b, 20-090 Lublin, Poland; katarzyna.k@vp.pl (K.K.); rush22235@gmail.com (F.W.); lukbry2@gmail.com (Ł.B.); b.teresinska2@gmail.com (B.T.); 3 Department of Machine Design and Mechatronics, Faculty of Mechanical Engineering, Lublin University of Technology, Nadbystrzycka 36, 20-618 Lublin, Poland; 4 Institute of Medical Sciences, The John Paul II Catholic University of Lublin, ul. Konstantynów 1H, 20-708 Lublin, Poland; 5 Department of Neurosurgery, Maria Sklodowska-Curie National Research Institute of Oncology, ul. W.K. Roentgena 5, 02-781 Warsaw, Poland; wojciech.w.czyzewski@gmail.com; 6 Department of Didactics and Medical Simulation, Medical University of Lublin, 20-954 Lublin, Poland

**Keywords:** Landau–Kleffner syndrome, neurosurgery, epilepsy, epileptic syndromes, aphasia

## Abstract

As speech-related symptoms of Landau–Kleffner syndrome (LKS) are often refractory to pharmacotherapy, and resective surgery is rarely available due to the involvement of the vital cortex, multiple subpial transection (MST) was suggested to improve patient outcome and preserve cortical functions. Here, we analyze the reports about MST use in LKS, regarding its impact on seizures, language, behavior, EEG, cognition, and reported adverse effects. In conditions like LKS, surgery is not a popular treatment option and presumably should be considered sooner. Candidates for MST should be selected carefully, optimally with the unilateral onset of epileptic activity. Laterality can be assessed using a methohexital suppression test (MHXT), electrical intracarotid amobarbital test, or magnetoencephalography. After MST, a significant percentage of LKS patients present seizure-free status, normalization of EEG patterns, and rapid behavior improvement. Data comprising language outcomes are mixed, with improvement reported in 23.8–100% of cases, and no superiority was found in the only study comparing MST with a non-surgical group. Cognitive outcomes are not well described. The risk linked to MST is described as low, with cerebral edema and transient neurological deficits being the most common complications. MST successfully improves seizure, EEG, and behavioral outcomes in LKS patients. However, its beneficial impact on language and cognition is not well proven. It is generally a safe neurological operation.

## 1. Introduction

Epilepsy is a widespread disability affecting around 50 million patients globally [1]. According to the International League Against Epilepsy (ILAE) [2], there are over 20 types of epilepsy syndromes, including epileptic encephalopathy with spike-and-wave activation in sleep (EE-SWAS) and developmental epileptic encephalopathy with spike-and-wave activation in sleep (DEE-SWAS). EE-SWAS and DEE-SWAS are uncommon syndromes. They comprise 0.5–0.6% of epileptic patients of all pediatric tertiary epilepsy centers [2]. The crucial criterion for their diagnosis is abnormal sleep EEG patterns. DEE-SWAS is recognized in patients with pre-existing disorders of the nervous system [2]. For example, it involves cognitive, language, behavioral, and motor dysfunctions present with spike-and-wave activation in sleep (SWAS). EE-SWAS concerns patients with incipiently normal development and subsequent activation of slow spike-and-wave complexes in non-rapid eye movement sleep (N-REM). Landau–Kleffner syndrome (LKS) is a specific EE-SWAS subtype that involves speech abnormalities.

LKS is a low-incidence epileptic encephalopathy [2]. It was first described in 1957 by William M. Landau and Frank R. Kleffner [3]. According to Muzio et al. [4], the occurrence of LKS is problematic to evaluate with a few hundred cases described globally and an estimated incidence of one in a million in a Japanese population. The disability concerns children from 2 to 8 years who had no previous manifestations [4]. Symptoms resemble autism, but LKS has a later age of onset and features auditory verbal agnosia, hyperkinetic behavior, difficulty with expressive language, and personality disorders [5]. The exact etiology of LKS is unknown. It can be correlated with a mutation in the gene glutamate ionotropic receptor NMDA type subunit 2A (*GRIN2A*) which codes one of the proteins from N-methyl-D-aspartate (NMDA) receptors, as dysfunctions of these receptors can cause similar clinical manifestations [4]. It is also possible that autoimmune factors are involved in the pathogenesis of LKS, as children with LKS present an increased rate of anti-brain-derived neurotrophic factor (BDNF) antibodies. Structural brain injuries are not related to its etiology [4].

Drug-resistant focal onset epilepsy has been operated on since 25 May 1886 [6]. The procedure consists of localizing the seizure focus and resecting part of the occupied cortex [7]. It was an efficient process but only if the focal did not involve any eloquent areas, including the primary speech, visual, motor, and sensory areas of the cortex. Resection of the eloquent cortex can lead to the decline of cognitive functions [7]. As a solution to this problem, a specified technique called multiple subpial transection (MST) was suggested [8]. Based on Morrell’s [8] investigation on monkeys, the transection of horizontal fibers while preserving columnar fibers protects the functional status of the cortex. MST relies on breaking the intracortical horizontal fiber system at 5mm intervals

The procedure is performed with a subpial transector, invented by Morrell et al. [8]. It has a form of springy stainless-steel wire with a blunt end pointed rightwards. The end of the transector is short (4 mm) [7,8] and angled at 105° [7] so the whole procedure does not exceed the gray matter [8].

**Figure 1:**
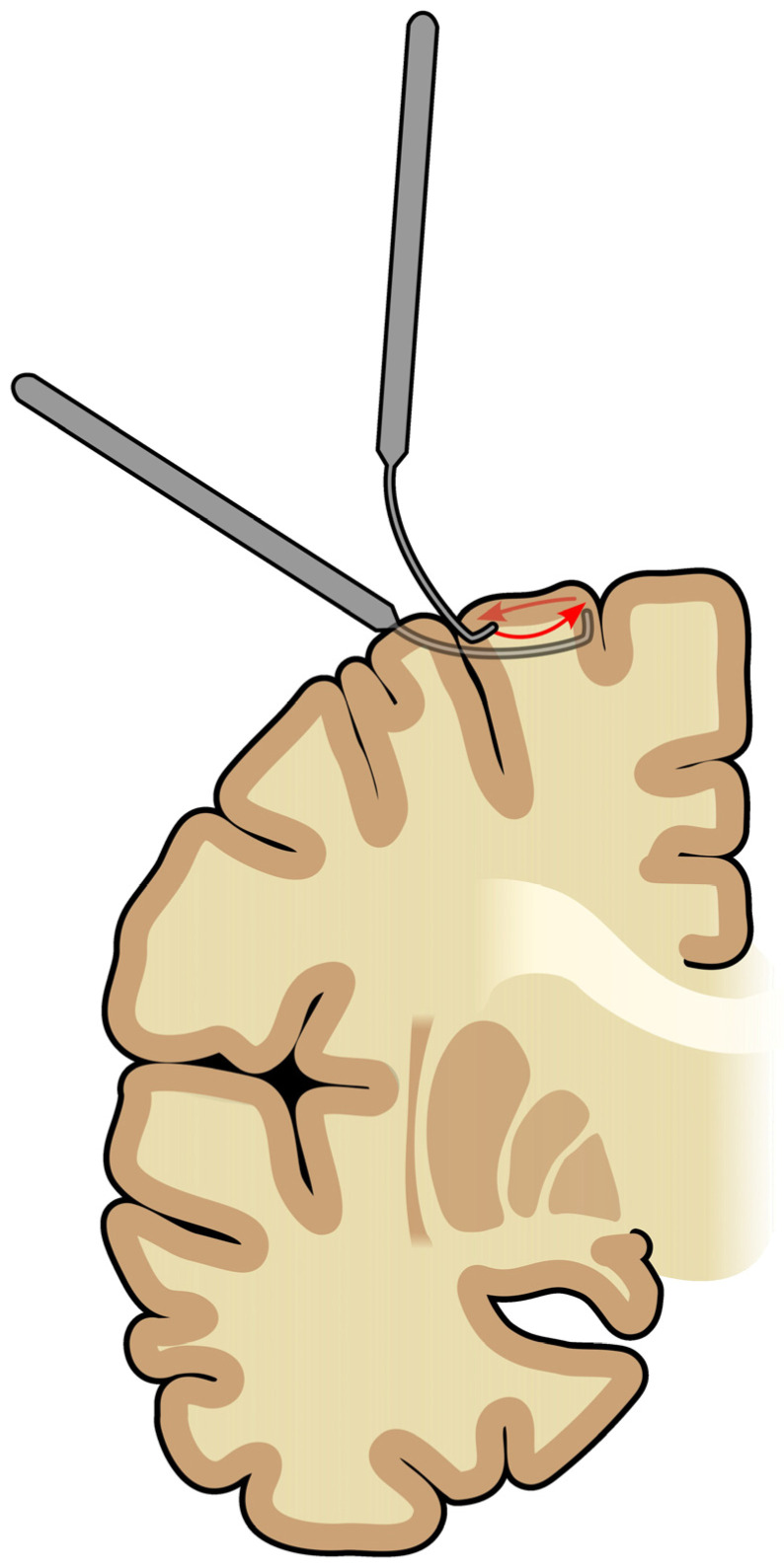
Illustration of the subpial transection procedure. The non-transparent transector and non-transparent arrow show the process of inserting the transector into the distal end of gyrus. The semi-transparent transectorshow the process of transecting fibers, and the semi-transparent shows its direction. The figure is based on a paper by Morrell et al. [8].

The procedure starts with shaving and immobilizing the patient’s head [9]. Afterward, the neurosurgeon performs a craniotomy, creating a large temporoparietal flap. An electrocorticography (ECoG) grid is placed on the cerebral cortex to monitor the epileptogenic discharges intraoperatively [9]. Then, a small incision in the pia is made with a point of #11 surgical blade [8,9], or a 20-gauge needle [7] maximally deep into the sulcus [8]. The transector is inserted via a pial opening and led underneath the gyrus until it reaches its far edge. The end is elevated until visible underneath the pia and pulled backward, across the gyrus (Figure 1). It is crucial not to damage the pia to avoid excessive bleeding and scarring [7]. The following transections are repeated 5 mm away until the ECoG shows cessation of epileptogenic activity [7,8,9]. Capillary bleeding at the site of insertion is common and is usually controlled with a thrombin-soaked sponge. The bleeding is presumably harmless and results in a thin red line, which helps to guide the next transections parallelly [8].

The main purpose of this article is to determine the effectiveness of MST in LKS treatment, regarding seizure outcomes, restoration of normal EEG patterns, and language, cognitive, and behavioral outcomes. We additionally included an aspect of MST safety. Due to the complexity of and updates to the epileptic syndromes nomenclature, we decided to use the nosological terms used by the cited authors. Where possible, we presented quantitative data regarding efficacy in the tables.

## 2. Review Methods

Due to heterogeneity in outcome measurement strategies, and epilepsy classifications, we decided to perform a narrative review.

**Figure 2:**
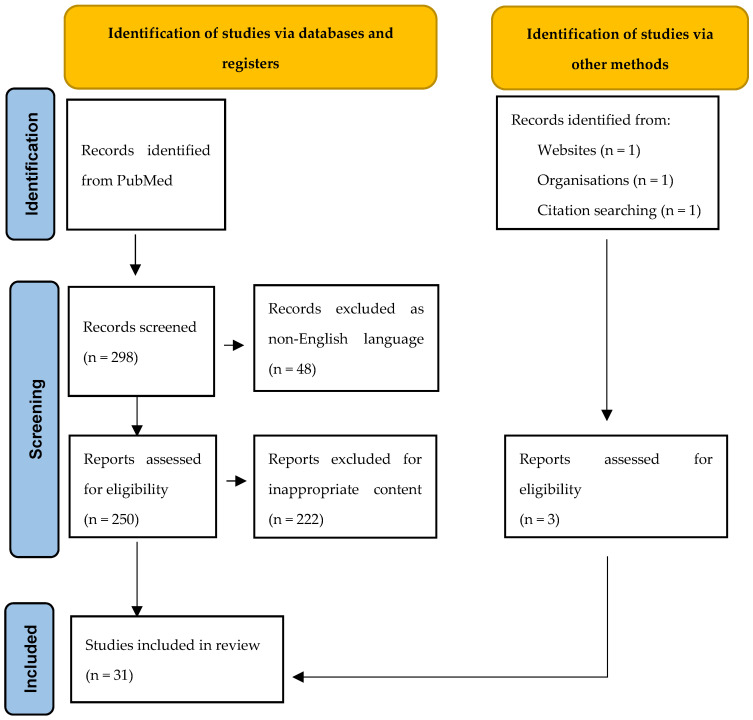
Flowchart illustrating the literature search. The flowchart is based on the PRISMA 2020 flow diagram for new systematic reviews which included searches of databases, registers, and other sources [10], modified.

We used the following search query: “Landau–Kleffner syndrome AND treatment”. Starting from 298 papers available on Pubmed, we excluded 48 papers not in the English language and a further 222 papers due to inappropriate content. We focused on papers describing MST procedures regarding patient qualification, efficacy, and safety. The efficacy was determined based on seizure, language, behavior, EEG, and cognitive outcomes. Additionally, to provide a wider context of LKS treatment, we included papers describing non-surgical LKS interventions. Ultimately, we collected 28 papers published between 1982 and 2023. Additionally, we added a paper from 1957 by Landau et al. [3] to describe LKS history, and a report from the UpToDate website and the World Health Organization (WHO) to provide more general information on epilepsy syndromes (Figure 2).

## 3. Overview of Treatment Options for LKS

Patients with LKS are offered several pharmacological treatment options, including antiepileptic drugs (AEDs), corticosteroids, benzodiazepines, and intravenous immunoglobulins [5,11,12].

As 70–80% of LKS patients suffer from seizures [13], they are often offered AEDs. However, there is a significant discordance between the severity of epilepsy and the severity of language loss [13]. Seizures are generally infrequent and respond to the antiseizure drugs quite well, while language function usually remains unaffected [12]. Of note, phenytoin, carbamazepine, and oxcarbazepine should not be used due to their ability to worsen electrical status epilepticus during sleep (ESES) [11,13].

One of the most commonly used drug groups is corticosteroids [13]. Compared with AEDs and benzodiazepines, they provide the best cognitive and EEG improvement in ESES patients [14]. They are also recommended in LKS and continuous spikes in slow wave sleep (CSWS) in the case of AED failure [15]. However, their use is limited due to their long-term use adverse effects [14]. What is more, after the cessation of steroids, seizures, speech abnormalities, and EEG outcomes relapse among the majority of patients [9,13,16]. Benzodiazepines are suggested as valuable alternatives to corticosteroids, as they provide better outcomes in EEG and cognition than AEDs among ESES patients [14]. Intravenous immunoglobulins can also be used, particularly if the patient is not responding to corticosteroids or the symptoms relapse after corticosteroid cessation [11,12]. Additionally, some reports suggest the beneficial effects of NMDA receptor antagonists among patients with *GRIN2A* mutations-related LKS [17].

Non-pharmacological therapies comprise ketogenic diets, vagus nerve stimulation, and surgical modalities, including MST [11,18].

Due to the rarity of LKS, randomized controlled trials are difficult to perform [13]. A 2020 systematic review found no complete randomized trials on pharmacological interventions, and data regarding surgery modalities are also limited [13,18].

## 4. Qualification for the MST Procedure

Surgery remains an uncommon treatment modality among patients with CSWS and presumably should be considered sooner in patients formerly diagnosed with ESES [11,14]. A 2014 survey among 232 neurologists in North America found that it was rarely preferred in the treatment of encephalopathies with CSWS, even regarding patients with structural lesions detected [19].

Cross et al. [20] suggested six criteria to be accomplished before qualification, including proper syndrome diagnosis, pharmacoresistance, adequate cost-benefit ratio, and confirmation of a focal and lateralized target [20].

In LKS, the usefulness of resective surgery is significantly limited due to the location of the epileptogenic zone, covering the Wernicke area [20,21]. To spare cortical function and limit pathological connectivity in the vital cortex, Morrell et al. [8] proposed MST. It was reported as a beneficial therapy for patients with LKS [11,12] and as the treatment of choice in cases with epileptogenic activity within the vital cortex [21]. However, candidates for MST should be carefully selected [11,21].

Morell et al. [22] suggested that unilateral epileptogenic zones may evoke a mirror epileptic activity in the homological contralateral cortex, which later may become independent [22]. Later, they showed that bilateral epileptogenic activity may disappear after unilateral MST, aiming for primary epileptogenic foci [23]. Thus, patients with unilateral onset of epileptogenic activity are suggested as the best candidates for MST [24]. To determine the laterality of epileptogenic foci in LKS patients, Morrell et al. [23] proposed a methohexital suppression test (MHXT, also: methohexitone suppression test [20,21,23]). In such tests, patients under EEG monitoring receive a dose of methohexital which suppresses the epileptic activity until it is limited to the epileptogenic foci [23]. Epileptogenic foci that are not autonomous are also suppressed. As patients experience transient apnea, the procedure must be performed under the care of an anesthesiologist. Another test suggested by Morrell et al. [23] to determine the laterality of epileptogenic foci is the electrical intracarotid amobarbital test (also: carotid amytal test [25], intracarotid sodium amytal and thiopentone suppression test [25,26]). The term “electrical” was suggested to distinguish it from the more popular Wada test for speech dominance [23]. The test relies on injection of amobarbital into the internal carotid artery, under EEG monitoring. If injected ipsilaterally, amobarbital inhibits spike–wave discharge in both hemispheres, whereas if injected contralaterally, the spike–wave activity is suppressed only on the site of injection [23]. The disadvantages of the MHXT and the electrical intracarotid amobarbital test are their invasiveness, which is particularly problematic in the pediatric population [21]. Nowadays, lateralization and localization are demonstrated via magnetoencephalography (MEG) [20].

## 5. Magnetoencephalography

MEG can show usefulness by indicating the unilateral nature of epileptogenic activity [21]. Diagnosis of LKS via MEG is strongly focused on latency differences between contralateral perisylvian areas. This parameter is poorly detected by EEG and may be crucial in determining the laterality of epileptogenic foci and thus qualification for MST [23,24]. In a study comprising 28 LKS patients, Paetau [24] found unilateral sole spike-propagating areas in 21% of patients, considering them as good candidates for MST [24].

MEG was also suggested as a tool for determining the chances of spontaneous recovery of linguistic functions. In a study of types of MEG patterns in two LKS patients, Castillo et al. [21] suggested that MEG showing reorganization in the Wernicke area is linked to spontaneous language recovery. Thus, those authors suggest that surgery should be additionally restricted to cases that show no signs of reorganization in MEG [21]. However, when searching for unilaterality, findings of the MHXT and MEG can be discordant in favor of MEG, as Paetau [24] showed in the case of a six-year-old LKS patient. The advantages of MEG extend to the avoidance of unnecessary surgeries. On the other hand, high costs and limited availability of MEG are its major disadvantages [24].

## 6. Seizure Outcomes

In LKS patients, seizures can subside spontaneously until adolescence [9,14] and have minor behavioral manifestations [23]. They are also satisfactorily controlled with pharmacotherapy [7,12,20,23]. However, surgical modalities including MST may also be effective [12,25]. Bermeo-Ovalle et al. [7] showed that MST provided Engel class I or II in up to 50% of LKS patients [7], and Morrell et al. [23] reported achieving seizure-free status in 79% of patients [23]. Even more beneficial results were described in a study comprising five pediatric LKS patients by Irwin et al. [25], as each patient achieved seizure-free status after three months of follow-up [25]. Of note, in the further follow-up, seizures relapsed in one of the patients but subsided soon after MST reoperation. Sawhney et al. [26], in a case series comprising three LKS patients with infrequent tonic–clonic seizures, reported substantial improvement in seizure status [26]. A single case of seizure freedom after MST was also reported by Neville et al. [27], in a case series of two epileptic patients, of whom one had LKS (Table 1) [27]. Nass et al. [28], in a cohort of seven LKS-related autistic epileptiform regression (AER) cases, noted an improvement in each patient with seizures, albeit usually transient [28].

## 7. Language Outcomes

It is generally stated that most LKS patients do not regain normal language skills [25], although the language function outcome is the most important result of surgery [23]. Language deterioration also is not proportionally dependent on EEG patterns: if the duration of aphasia exceeded one year, steroids provide no beneficial effects, despite normalization of the EEG [13]. Neville et al. [27] described a single LKS case of a patient showing no understanding of language. After MST, the patient presented partial verbal improvement, using two to three words, and elaborated a word reading vocabulary [27]. Morrell et al. [23] noticed language improvement in 11 of 14 preoperatively mute pediatric patients, of whom seven presented normal speech and four showed marked improvement [23]. The language recovery was preceded by the return of sign language and was gradual, with the first words reported about 12 weeks post-operatively. Grote et al. [29] conducted another study of 14 LKS patients, of whom 10 patients were originally described in 1995 by Morrell [23]. Studying receptive and expressive vocabulary separately, they found significant improvement in seven and eight patients, respectively [29]. Considering data of receptive and expressive vocabulary tests cumulatively, four children improved in both tests, six improved in one test with no changes in the second, one improved in one test while worsened on the second, two children presented no changes on both tests, and one worsened in one test with no significant changes on the second. A longer follow-up was positively correlated with better receptive and expressive outcomes, whereas longer illness before surgery was inversely correlated with better receptive vocabulary [29]. Conversely to Morrell et al. [23], those authors suggested that language improvement was a matter of years, rather than months [29]. Ovalle et al. [7], sharing their experience of 24 LKS patients, stated that after six months of follow-up, two-thirds of the children who underwent perisylvian MST achieved significant improvement in language function [7]. What is more, such patients were able to speak in complex sentences, and nine of them experienced total language recovery. Language improvement with one case of fluent speaking was also noted by Sawhney et al. [26] in a group of three previously mute LKS patients (Table 2) [26]. Language improvement, albeit moderate and not universal, was also noted among AER patients, with a preference towards receptive rather than expressive functions [28].

On the other hand, Irwin et al. [25] observed that among five LKS patients, no child regained age-appropriate language functions despite follow-up as long as six years after the operation [25]. Based on Grote et al.’s [29] premise that longer follow-up is a predictor of better language outcome and no available research comparing MST with a control group, Irwin et al. [25] suggested that the speech improvement may be attributed to LKS natural history rather than MST [25]. To test this hypothesis, Downes et al. [30] for the first time compared the efficacy of MST (n = 14) with a control group (n = 21) in a cohort comprising LKS and more global ESES-related regression patients [30]. Those authors showed no statistically significant differences in language abilities and parent-reported quality of life between surgical and non-surgical groups. Thereafter, Kheder et al. [31] suggested that the results may be influenced by adding ESES-related regression, and outcomes of LKS patients should be analyzed separately [31]. However, subgroup analysis confirmed no differences between surgical and non-surgical groups in both LKS and ESES-related regression subgroups [32].That was a rationale for a statement that MST in LKS and ESES-related regression does not provide worthwhile benefits, compared with patients who do not undergo surgery [30,32].

## 8. Behavior Outcomes

Behavioral abnormalities linked with LKS include decreased attention span, hyperactivity, and aggressive oppositionality [25]. Similar to seizures, behavioral changes in LKS are generally controlled with AEDs and immunotherapy [7], but MST also can provide significant behavior improvements [25]. Neville et al. [27] reported the case of an LKS patient who became more interactive and focused, showed more understanding of a situation, and was more hyperactive within a few days after MST (Table 3) [27]. Nass et al. [28] in a study comprising seven AER patients reported moderate behavior progress including prolonged attention spans, increased eye contact, rarer maladaptive behaviors, and increased ability to focus [28]. Addressing the issue of behavior indirectly, Morrell et al. [23] described the use of MST in a cohort of 14 pediatric patients, of whom 50% were able to study in a normal school post-operatively [23]. Similarly, Grote et al. [29], in a cohort partially overlapping with those of Morrell [23], stated that four of 12 evaluated patients were able to attend mainstream classrooms with no special service needed [29]. Some authors even stated that irrespectively of the extent of language improvement, MST is justified as a powerful tool to improve behavior immediately, which reflected the invoked opinion of one LKS patient’s parents [25,26].

## 9. EEG Outcomes

Similarly to clinical seizures, EEG features of ESES can spontaneously appear until adolescence [9]. Irwin et al. [25] noted that MST eliminated ESES or SWAS and regained normal background activity in each of five LKS cases, while Morrell et al. [23] described normalization of EEG in nine of 14 LKS patients. Less beneficial results were reported by Cross et al. [20], with a minority of patients managing to reach EEG normalization (Table 4).

## 10. Cognitive Outcomes

Similar to language skills, the cognitive benefits of MST in LKS patients remain uncertain [14,25]. Irwin et al. [25] detected no statistically significant differences in his five LKS patients, whereas one patient improved from a non-testable intelligence quotient (IQ) level to being a cooperative and testable child [25].

## 11. Complications

The risk level of MST is considered low [20]; however, as a neurosurgical procedure, MST is expected to inflict some acute adverse effects. One of them is cerebral edema, which is most prominent on the third or fourth day after surgery [7]. Some transient deficits in transected cortical function may also appear. Usually, they resolve spontaneously within two to three weeks, although mild deficits may sometimes persist for months. Chronic cognitive deficits may also appear [7]. In a study by Bermeo-Ovalle et al. [7], 7% of MST epileptic patients experienced permanent deficits in the form of foot drop, language deficit, parietal sensory loss, and attenuation of rapid skilled movements, and also experienced extra-axial collection [7,20]. Morrell et al. [23] in a group of 74 epileptic patients reported two cases of functional deterioration, of which one was linked to a small infraction and the second was related to a hemorrhage of a white matter [23]. Among MST-related morbidities, other authors reported meningitis, CSF leakage, and strokes [29,30].

## 12. Conclusions

Given the rarity of LKS and its limited representation in the literature, MST appears not to be a popular treatment modality. The success of the procedure has been suggested to depend significantly on adequate patient qualification and thus availability of advanced diagnostic methods such as MEG. It appears to be particularly effective as an early intervention in cases of progressive aphasia. This review highlights MST as a potential option for improving seizure control, EEG normalization, and behavioral outcomes, with variable effects on language and cognition.

MST is generally described as a safe neurological procedure. Its low complication rates and behavioral benefits could support its integration into multidisciplinary care of LKS patients, although its language benefits are disputable. Still more data are needed to evaluate MST serviceability, especially given that most recent findings do not support its beneficial impact on language function. Further research should prioritize long-term, multi-center studies on MST’s efficacy in language recovery, comparison with non-surgical interventions, and exploration of adjunctive therapies.

## Figures and Tables

**Table 1:** Seizure control after MST in LKS patients.

Number of LKS Patients	Time of Assessment	Engel Class I	Engel Class II	Engel Class III	Engel Class IV	Seizure Improvement, Not Specified	Surgical Procedures, % of Patients	Additional Information	Authors
10	N/A	N/A	N/A	N/A	N/A	50%	MST, not detailed	-	Cross [20]
12	13–78 months	75% *	N/A	N/A	N/A	-	MHXT (100% *); MST of Wernicke (100% *) and Broca (N/A) area; Temporal lobectomy (16.7% *)	Two additional patients had no evidence of seizures (not included). Seizure-relapse patients (25%) achieved no benefit in the language domain.	Morrell [23]
5	Post-surgery	60%	40% *	0% *	0% *	-	Carotid amytal and thiopentone suppression tests (N/A) MST, usually of superior and middle temporal gyri	-	Irwin [25]
5	At the latest available follow-up	100%	0%	0%	0%	-		After seizure relapse, one of the patients was reoperated on and regained Engel I class.	
3	N/A	66.7% *	N/A	N/A	N/A	-	Carotid amytal test; extensive MST ** of precentral gyrus, sylvian area, and posterior temporal and parietal lobes (33.3% *). MST ** (66.7% *)	-	Sawhney [26]
1	6 months	100% *	0%	0%	0%	-	MST, not detailed	-	Neville [27]

Abbreviations: LKS—Landau–Kleffner syndrome; MHXT—methohexital suppression test; MST—multiple subpial transection; N/A—not available. *—Value calculated or an adequate Engel class determined, based on data from publication; **—according to description by Morrell et al. [8].

**Table 2:** Language outcomes after MST in LKS patients.

Number of LKS Patients	Time of Assessment	Language Function Improvement, %	Regaining Normal Language Function, %	Surgical Procedures, % of Patients	Additional Information	Authors
10	N/A	70%	0%	MST, not detailed	-	Cross [20]
14	13–78 months	79%	50%	MHXT (92.9% *); MST of Wernicke (100% *) and Broca (N/A) area; temporal lobectomy (14.3% *)	-	Morrell [23]
5	Post-surgery	100%	0%	Carotid amytal and thiopentone suppression tests (N/A); MST ***, usually of superior and middle temporal gyri	-	Irwin [25]
5	At the last available follow-up	100%	0%			
3	N/A	100%	N/A	Carotid amytal and thiopentone test; extensive MST ** of precentral gyrus, sylvian area, and posterior temporal and parietal lobes (33.3% *); MST ** (66.7% *)	-	Sawhney [26]
1	-	100%	-	MST, not detailed	-	Neville [27]
13	0.6–6.6 years	53.85% * ^a^	N/A	MHXT (N/A); MST: fronto-temporo-parietal (61.5% *), fronto-temporal (15.4% *); temporo-parietal (15.4% *), and temporal (7.7% *)	We excluded one patient from calculations due to lacking post-operative PPVT-R measure	Grote [29]
14	0.5–6.6 years	57.14% * ^b^	N/A	MHXT (N/A); MST: fronto-temporo-parietal (57.1% *), fronto-temporal (14.3% *); temporo-parietal (21.4% *), and temporal (7.1% *)	-	
13	-	23.08% *	7.7% *	MHXT (N/A) and/or MEG (N/A); MST **, posterior temporal region	-	Downes [30]

Abbreviations: LKS—Landau–Kleffner syndrome; MEG—magnetoencephalography; MHXT—methohexital suppression test; MST—multiple subpial transection; N/A—not available; PPVT-R—Peabody Picture Vocabulary Test-revised; EOWPVT-R—Expressive One Word Picture Vocabulary Test-revised. *—Value was calculated based on data from publication; **—according to description by Morrell et al. [8]; ***—according to description by Morrell et al. [8] (and with original transectors in most cases); ^a^—receptive vocabulary was measured with PPVT-R; ^b^—expressive vocabulary was measured with EOWPVT-R.

**Table 3:** Behavior outcomes after MST in LKS patients.

Number of LKS Patients	Time of Assessment	Behavioral Improvement	Surgical Procedures, % of Patients	Authors
10	N/A	10%	MST, not detailed	Cross [20]
5	Post-surgery	100%	Carotid amytal and thiopentone suppression tests (N/A); MST *, usually of superior and middle temporal gyri	Irwin [25]
5	At the last available follow-up	100%		
1	6 months	100%	MST, not detailed	Neville [27]

Abbreviations: LKS—Landau–Kleffner syndrome; MST—multiple subpial transection; N/A—not available; *—according to description by Morrell et al. [8] (and with original transectors in most cases).

**Table 4:** EEG outcomes after MST in LKS patients.

Number of LKS Patients	Normal EEG Activity	Surgical Procedures %, of Patients	Additional Information	Authors
10	30%	MST, not detailed	-	Cross [20]
14	64%	MHXT (92.9% *); MST of Wernicke (100% *) and Broca (N/A) area; temporal lobectomy (14.3% *)	3 of 5 patients with recurrent EEG abnormalities presented a recurrence of clinical seizures	Morrell [23]
5	100%	Carotid amytal (N/A) and thiopentone suppression tests (N/A). MST *, usually of superior and middle temporal gyri	-	Irwin [25]

Abbreviations: EEG—electroencephalography; LKS—Landau–Kleffner syndrome; MHXT—methohexital suppression test; MST—multiple subpial transection; *—according to description by Morrell et al. [8] (and with original transectors in most cases).

## Abbreviations

AEDs	Antiepileptic drugs
AER	Autistic epileptiform regression
BDNF	Brain-derived neurotrophic factor
CSWS	Continuous spikes in slow wave sleep
DEE-SWAS	Developmental epileptic encephalopathy with spike-and-wave activation in sleep
ECoG	Electrocorticography
EE-SWAS	Epileptic encephalopathy with spike-and-wave activation in sleep
EOWPVT-R	Expressive One Word Picture Vocabulary Test-revised
ESES	Electrical status epilepticus during sleep
GRIN2A	Glutamate Ionotropic Receptor NMDA Type Subunit 2A
ILAE	International League Against Epilepsy
IQ	Intelligence quotient
LKS	Landau–Kleffner syndrome
MEG	Magnetoencephalography
MHXT	Methohexital suppression test
MST	Multiple subpial transection
N/A	Not available
NMDA	N-methyl-D-aspartate receptor
N-REM	Non-rapid eye movement sleep
PPVT-R	Peabody Picture Vocabulary Test-revised
SWAS	Spike-and-wave activation in sleep
WHO	World Health Organization
